# Editorial Closing: Plant Biotic and Abiotic Stresses Special Issue

**DOI:** 10.3390/life16050742

**Published:** 2026-04-29

**Authors:** Gastón Alfredo Pizzio

**Affiliations:** 1 CIT Río Negro, Universidad Nacional de Río Negro, Viedma CP8500, Río Negro, Argentina; gapizzio@gmail.com; 2 Institute for Integrative Systems Biology (I2SysBio), Universitat de València-CSIC, 46908 Paterna, Spain

## 1. Introduction

In the current Anthropocene era, the exacerbation of climate volatility has transcended mere academic concern to become an existential threat to global food security. Global agriculture faces an unprecedented convergence of challenges [1]. Climate change is intensifying the frequency and severity of drought, salinity, and temperature extremes, while the mounting pressure of pathogens and herbivores continues to threaten crop productivity worldwide [2]. At the heart of plant adaptive responses to these combined pressures lies a sophisticated and remarkably dynamic molecular language—one in which small signaling molecules orchestrate large-scale physiological, molecular, and developmental outcomes. The use of different biostimulant formulations, which modulate this plant molecular language and strengthen tolerance mechanisms to stress, represents a promising strategy for addressing this growing threat [3]. These formulations, ranging from phytohormones to antioxidants, have the potential to enhance plant resilience against stress through processes such as osmotic adjustment, antioxidant response stimulation, and immune system activation, among others. Therefore, the development of new strategies in crop management to improve tolerance to both biotic and abiotic stresses is fundamental to reducing production losses and increasing the adaptive capacity of crops against different stress factors.

This Special Issue was conceived with the conviction that advancing our understanding of the molecular mechanisms underlying plant stress responses is not merely an academic pursuit, but an urgent scientific imperative with direct implications for food security, ecosystem sustainability, and agricultural resilience in a rapidly changing world.

The 25 contributions gathered in this Special Issue—comprising 23 research articles and 2 reviews—collectively illuminate how plants, from model species to staple crops and native vegetation, deploy, integrate, and fine-tune diverse molecular mechanisms, including hormonal signaling networks, to navigate adversity. Together, they span a broad range of biological scales, from genome-wide gene family characterization and leaf-level physiological traits to whole-plant agronomic performance and microbe-mediated stress alleviation, reflecting the genuine interdisciplinarity that the field demands.

## 2. Overview of Relevant Contributions and Key Advances

This Special Issue brought together research spanning multiple-crop and non-crop systems, stress types, and methodological approaches from research groups across the globe, offering a rich and complementary picture of how plants and their associated organisms respond to environmental adversity.

### 2.1. Water Deficit and Drought Stress

One of the central themes emerging from this collection is the critical importance of water deficit and drought as a primary constraint on crop productivity. De Oliveira et al. addressed this challenge directly by evaluating the drought resilience of 43 Latin American popcorn lines (*Zea mays* L. var. *everta*) under contrasting water regimes. Water deficit drastically reduced grain yield, popcorn volume, and chlorophyll content but increased anthocyanins as an adaptive response. Lines L292 and L688 stood out as the most tolerant, proving to be outstanding candidates for drought-tolerant breeding programs [4].

Expanding the perspective beyond crop species, Tan et al. investigated the leaf hydraulic and economic trait networks of 19 temperate desert shrub species in Inner Mongolia, a system subjected to chronic arid stress. Their findings revealed that these shrubs adopt a predominantly “tolerance” hydraulic strategy, maintaining leaf turgor even under severe embolism conditions—a strategy mechanistically dependent on the negative turgor loss point values observed across species. Notably, the study found no trade-off between hydraulic efficiency and embolism resistance, a departure from patterns reported in less water-limited ecosystems, and identified leaf thickness as the central hub trait linking hydraulic safety, carbon investment, and anatomical structure. This contribution provides essential physiological context to understand the anatomical and biophysical basis in resilient native species and offers a blueprint for translational research in crops [5].

### 2.2. Salinity Stress

Among the most prevalent abiotic stressors affecting modern agricultural systems, soil salinity represents a critical limiting factor for crop growth, development, and productivity. Malik et al. demonstrated the considerable potential of arbuscular mycorrhizal fungi (AMF) symbiosis in alleviating salinity stress in *Cenchrus ciliaris*, a drought-resistant forage grass of critical importance to arid rangelands in Saudi Arabia and across the Arabian Peninsula. A consortium of four AMF species (*Claroideoglomus etunicatum*, *Funneliformis mosseae*, *Rhizophagus fasciculatum*, and *R. intraradices*) consistently improved shoot and root morphology, photosynthetic pigment accumulation, and the biosynthesis of proline and phenolic compounds under NaCl concentrations up to 300 mM. Critically, the AMF colonization rate itself declined with increasing salinity, yet the symbiosis remained functional and growth-promoting even under severe stress—a testament to the remarkable plasticity of the mycorrhizal relationship. The enhanced proline and phenolic accumulation observed in AMF-inoculated plants is consistent with AMF-mediated modulation of jasmonic acid and salicylic acid pathways, reinforcing the view that beneficial microorganisms act, at least in part, through the same hormonal circuits that govern endogenous plant stress responses [6].

Elekou and coworkers investigated how saline stress affects the growth and genetics of two avocado varieties, finding that the americana breed possesses a higher salt tolerance than the sensitive drymifolia variety. Researchers identified and modeled the PaHAK2 gene, a high-affinity potassium transporter that plays a critical role in maintaining osmotic balance during the early stages of plant development. Experimental results show that while salt delays germination speed, the americana variety exhibits a significant increase in PaHAK2 expression, providing an adaptive advantage under high salinity. The research concluded that this specific gene is essential for seedling establishment and could serve as a vital marker for breeding salt-resistant crops. Such findings are crucial for improving agricultural yields in regions where soil salinity increasingly limits the cultivation of sensitive fruit trees [7].

### 2.3. Temperature-Driven Stress

Temperature is one of the most critical environmental factors governing plant growth, development, and productivity, as it directly influences a wide array of physiological, biochemical, and molecular processes essential for plant survival. Both extreme heat and freezing temperatures represent major abiotic stressors that can severely compromise plant performance, causing irreversible damage at the cellular and tissue levels. Understanding and enhancing plant thermotolerance has become a research priority of paramount importance for ensuring global food security.

Li et al. analyzed eighteen CBF gene family members in the kiwifruit species *Actinidia arguta* to understand their role in low-temperature stress responses. The investigation specifically focused on AaCBF4.1, which showed significant expression in roots and dormant branches and was highly induced by chilling. When this gene was overexpressed in Arabidopsis, the transgenic plants exhibited superior cold tolerance, characterized by higher chlorophyll levels and reduced oxidative damage. Ultimately, these findings provide a genetic foundation for breeding cold-resistant kiwifruit varieties through the ICE-CBF-COR signaling pathway [8].

Liu and coworkers explored how long non-coding RNAs (lncRNAs) regulate the response of the evergreen shrub *Rhododendron delavayi* to extreme heat. Using transcriptome sequencing, the authors identified over a thousand differentially expressed lncRNAs and thousands of associated genes that fluctuate when the plant is exposed to high temperatures. The study focused on cis- and trans-regulatory mechanisms, illustrating how these non-coding transcripts influence both neighboring and distant protein-coding genes. KEGG enrichment analysis linked these genetic interactions to vital pathways such as phenylpropanoid biosynthesis and amino acid metabolism. Ultimately, the researchers highlighted three specific target genes—TrxG, PEPC, and CCR—as essential candidates for future genetic engineering. These findings provide a theoretical framework for developing more heat-tolerant ornamental plants through targeted molecular improvement [9].

Interestingly, a research article developed a systematic screening platform to evaluate how 27 native Korean woody plant species respond to the combined challenges of sequential drought and heat stress. By applying a protocol of two-week water deprivation followed by extreme temperature exposure, the authors used physiological markers like relative water content and electrolyte leakage to measure hydration and membrane stability. Through additional cellular and phenotypic analysis, the study identified high-tolerance species such as *Chamaecyparis obtusa* and various Quercus species, while noting the extreme vulnerability of the silk tree. The findings offer a robust framework for selecting climate-resilient trees, which is essential for successful reforestation and urban forestry in an era of global warming. This multiscale evaluation proves that the synergy of multiple stressors requires more complex assessment than studying drought or heat in isolation [10].

### 2.4. Biotic Stress

Plants are continuously exposed to a wide range of biotic stressors throughout their life cycle, including pathogenic microorganisms such as fungi, bacteria, and viruses, as well as herbivorous insects and parasitic nematodes, among others. These biotic agents can cause significant damage to plant tissues and organs, disrupting fundamental physiological and biochemical processes such as photosynthesis, nutrient uptake, and hormonal signaling, ultimately leading to substantial reductions in crop yield and quality. On this matter, two articles were contributed, addressing plant resistance to biotic stress, albeit from different perspectives: one through antimicrobial protein engineering and the other through the phenotypic screening of germplasm, with direct implications for plant breeding.

Meng et al. explored the role of chicken egg white lysozyme (*Gallus gallus*, EWL) as a resistance agent against pathogens in *Nicotiana benthamiana*. Experimental results showed differentiated effects depending on the type of pathogen. Against *Turnip mosaic virus* (TuMV), EWL overexpression significantly increased viral accumulation, suggesting that the protein could interfere with the plant’s antiviral defense mechanisms, paradoxically facilitating infection. In contrast, no significant effect was observed on infection by *Tobacco mosaic virus* (TMV), indicating virus-specificity. Against the necrotrophic fungus *Botrytis cinerea*, plants expressing EWL showed a significant reduction in the area of necrotic lesions 9 days post-inoculation, demonstrating greater fungal resistance. The authors attribute this effect to EWL’s ability to hydrolyze β-1,4-glycosidic bonds in fungal cell walls. The findings point to the biotechnological potential of EWL as a bioengineering tool for developing varieties with greater fungal resistance, although they caution that its impact on viral infections requires further research to understand the mechanisms involved [11].

Yu and coworker performed a systematic evaluation of the resistance of 24 alfalfa (*Medicago sativa*) cultivars to the southern root-knot nematode *Meloidogyne incognita*, one of the most destructive pathogens in global agriculture. The results identified wide differences in resistance among the evaluated materials. The Chinese variety Gannong No. 9 stood out as highly resistant. The American varieties Dryland and Moste also showed remarkable resistance. At the opposite end of the spectrum, Nongjing No. 1 (China) and Catera (France) were classified as susceptible. The authors propose the use of this type of study as a standard framework for resistance evaluation in breeding programs [12].

Taken together, the 25 contributions to this Special Issue have made meaningful progress in addressing several persistent gaps in our understanding of crop resilience mechanisms. First, they reinforce the value of integrating multiple levels of biological organization—from molecular gene networks to whole-plant physiology to ecosystem-level microbiome interactions—in building a comprehensive picture of stress adaptation. Second, they collectively highlight the centrality of hormone-responsive cis-regulatory elements and osmolyte metabolism as convergence points between biotic and abiotic signaling, even in studies not explicitly framed around phytohormones. Third, the inclusion of both model and non-model species, as well as native vegetation, reflects a maturing field that no longer confines fundamental discoveries to a handful of laboratory systems.

## 3. Future Directions

Despite the advances documented in this collection, important questions remain open and should guide future research priorities. The hormonal crosstalk underlying simultaneous biotic and abiotic stress responses—a scenario of increasing relevance under ongoing climate change—remains insufficiently characterized at the mechanistic level, particularly in crop species beyond *Arabidopsis* and rice. The role of epigenetic regulation and transgenerational stress memory in modulating hormonal responses deserves considerably more attention, especially in the context of breeding for durable resilience. Similarly, the contribution of the root microbiome—including AMF and plant growth-promoting bacteria—to hormonal homeostasis under field conditions remains largely unexplored, as most studies, including several in this collection, were conducted under controlled environments. Future research should prioritize multi-omics integration, combining transcriptomics, metabolomics, and hormone profiling across spatial and temporal scales, to map the full signaling landscape connecting environmental perception to adaptive output. Finally, translating mechanistic discoveries into practical breeding strategies—whether through marker-assisted selection, precision genomic editing of hormone pathway genes, or microbiome engineering—remains the critical and underserved bridge between fundamental plant science and tangible agricultural impact.

## 4. Conclusions

The research presented in this Special Issue collectively affirms that phytohormones and the molecular networks they govern remain among the most promising levers for enhancing crop resilience to the complex and compounding stresses of our time. Whether operating through the regulation of osmotic adjustment, the orchestration of defense responses, the modulation of root architecture, or the mediation of beneficial symbiotic associations, these small signaling molecules continue to exert key biological effects—and the careful elucidation of their language represents one of the most rewarding and consequential frontiers in contemporary plant biology. We extend our sincere gratitude to all contributing authors, reviewers, and the editorial team of *Life* (MDPI) for their commitment and dedication to advancing this vital field of research.
